# The Use of Biological Meshes in Diaphragmatic Defects – An Evidence-Based Review of the Literature

**DOI:** 10.3389/fsurg.2015.00056

**Published:** 2015-10-21

**Authors:** Stavros A. Antoniou, Rudolph Pointner, Frank-Alexander Granderath, Ferdinand Köckerling

**Affiliations:** ^1^Center for Minimally Invasive Surgery, Neuwerk Hospital, Mönchengladbach, Germany; ^2^Department of General Surgery, University Hospital of Heraklion, Heraklion, Greece; ^3^Department of General and Visceral Surgery, Hospital Zell am See, Zell am See, Austria; ^4^Department of Surgery, Center for Minimally Invasive Surgery, Vivantes Hospital, Berlin, Germany

**Keywords:** biologic mesh, biologic graft, hiatal hernia, diaphragmatic rupture, paraesophageal hernia, fundoplication

## Abstract

The widespread use of meshes for hiatal hernia repair has emerged in the era of laparoscopic surgery, although sporadic cases of mesh augmentation of traumatic diaphragmatic rupture have been reported. The indications for biologic meshes in diaphragmatic repair are ill defined. This systematic review aims to investigate the available evidence on the role of biologic meshes in diaphragmatic rupture and hiatal hernia repair. Limited data from sporadic case reports and case series have demonstrated that repair of traumatic diaphragmatic rupture with biologic mesh is safe technique in both the acute or chronic setting. High level evidence demonstrates short-term benefits of biologic mesh augmentation in hiatal hernia repair over primary repair, although adequate long-term data are not currently available. Long-term follow-up data suggest no benefit of hiatal hernia repair using porcine small intestine submucosa over suture repair. The effectiveness of different biologic mesh materials on hernia recurrence requires further investigation.

## Introduction

Blunt or penetrating trauma of the abdomen and thorax may cause injury to the diaphragm (1). In the case of traumatic diaphragmatic rupture, abdominal organs such as the stomach, spleen, colon, or the liver may herniate into the thoracic cavity causing a wide range of symptoms, which may occur several years after the injury (2–5). Chest X-ray is often diagnostic, whereas computed tomography and magnetic resonance imaging provide detailed information about the herniated structures and the size of the defect (6, 7). There is no consensus on the absolute indications for surgery or the timing of surgical intervention. A traumatic rupture of the diaphragm is generally considered an indication for surgical repair, especially in the presence of symptoms.

Relevant literature evidence is limited, mainly due to the rarity of the condition. Primary suture repair or covering the defect with a synthetic mesh has been the standard of care during the past decades (8). Biologic meshes have been thought to be effective in closing the diaphragmatic defect, induce limited inflammatory response, and minimize adhesion formation.

In the presence of insufficient evidence, there is ongoing debate on the need of augmentation of the diaphragmatic hiatus during hernia repair (9). A number of randomized controlled trials (RCTs) and a meta-analysis have demonstrated lower recurrence rates after mesh repair; however, long-term data are not currently available (10). Several studies have reported complications, which has created skepticism with regard to the benefits of augmented hiatal hernia repair (11–13).Several biologic materials have been manufactured and are currently in use in surgical practice. Experimental data have shown biologic meshes to possess characteristics of an ideal mesh material, such as reduced adhesion formation, improved biocompatibility, decreased inflammatory response, and optimal neovascularization (14). Our objective was to review the evidence investigating the role of biologic meshes in traumatic repair of the diaphragm and in hiatal hernia repair.

## Materials and Methods

### Repair of Traumatic Diaphragmatic Defects

Electronic searches of the Medline database were conducted using the PubMed search engine. The following combination of terms and keywords was applied: (trauma OR traumatic OR posttraumatic OR rupture*) AND (diaphragm* OR phren*) AND (mesh OR implant). The search returned 141 reports. The last search was run in November 2014. Titles and abstracts were interrogated and clinical reports on the use of biologic material for closure of traumatic diaphragmatic defects were selected. The full texts of 17 articles were assessed for eligibility; three relevant reports were identified (15–17). The remaining 15 articles were excluded because they reported on the use of synthetic materials in diaphragmatic rupture repair or did not provide relevant outcomes. A summary of the study characteristics and outcomes is presented in Table 1.

**Table 1:** **Characteristics and outcomes of studies reporting on repair of traumatic diaphragmatic rupture with the use of biologic mesh**.

References	Study design	Patient characteristics	Mesh material	Intervention details	Follow up	Outcome	Conflict of interest	LoE^a^
Teicher et al. (15)	Case report	25 years old Acute case Grade IV left-sided diaphragm rupture	HADM	Open tension-free repair with a 4 cm × 4 cm mesh Anchorage with a 3–0 polydioxanone running suture	6 months Chest X-ray	No recurrence	NR	5
Pulido et al. (16)	Case report	70 years old Chronic case Accident 41 years before – no surgery Inflamed gallbladder and small bowel herniated	HADM	Laparoscopic cholecystectomy Anchorage with interrupted #0 polyethylene sutures	NR	Empyema, bile leak, and biliary effusion of the right pleura ERCP and VAT pleurodesis	NR	5
Al-Nouri et al. (17)	Case series	*n* = 4 2 right-sided, 2 left-sided diaphragm ruptures 3 chronic cases, 1 acute case 1 case of concurrent pleural empyema	HADM/SIS	Thoracotomy or thoracotomy/laparotomy repair Suture approximation and mesh reinforcement Pleurodesis in the case of pleural empyema	1–2 years Chest X-ray	No recurrence	NR	4

*HADM, human acellular dermal matrix; SIS, small intestine submucosa; ERCP, endoscopic retrograde cholangiopancreatography; VAT, video-assisted thoracoscopy; LoE, level of evidence*.

*^a^Based on the Oxford Centre for Evidence-based Medicine – Levels of Evidence (March 2009)*.

### Hiatal Hernia Repair with Mesh Augmentation

Similarly, Medline was searched to identify relevant clinical evidence using the PubMed interface up to November 2014. The keywords (hiat*) AND (hernia) AND (mesh OR implant) were used. Of a total of 309 records, 28 articles were selected for full text review based on relevant information from titles and abstracts. Twenty-two articles provided relevant outcome data on mesh-reinforced hiatal hernia repair with biologic meshes (18–39). The study characteristics and outcomes are listed in Table 2.

**Table 2:** **Characteristics and outcomes of studies reporting on hiatal hernia repair with the use of biologic mesh**.

References	Study design	Patient characteristics	Mesh material	Intervention details	Follow up	Outcome	Conflict of interest	LoE^a^
Oelschlager et al. (18)	Retrospective case series	*n* = 9 Type III hernia, *n* = 8 Type II hernia, *n* = 1 Median age 63 years (range 47–80)	SIS	Keyhole or *U*-shaped SIS 7 cm × 10 cm mesh anchored with interrupted silk sutures Nissen fundoplication and gastropexy	3–16 months UGIS ± UGIE	1 recurrence 1 need for dilatation for mild persistent dysphagia	Yes	4
Strange (19)	Retrospective case series	*n* = 12 Patients with “large hiatal defects” Median age: 66 years	SIS	Suture repair Keyhole mesh, circular portion 2.5–3 cm anchored with #2–0 non-absorbable sutures fixed to the esophagus	Median 11 months UGIS	No recurrence	NR	4
Johnson et al. (20)	Case report	Type III, 82 years old Type IV, 62 years old Second recurrence, 53 years old	HACD	Suture repair with interrupted non-absorbable sutures Onlay mesh placement Nissen fundoplication	UGIS in the early postoperative period Symptom outcome at 8–10 months	No early recurrence Lack of symptoms at follow up	NR	5
Oelschlager et al. (21–23)	Assessor-blinded RCT	*n* = 108 Symptomatic paraesophageal hernia size >5 cm	SIS	Suture repair with interrupted #2–0 or #0, *n* = 57 U-shaped 7 cm × 10 cm mesh anchored with interrupted sutures, additionally to the suture repair, *n* = 51 Nissen fundoplication	Short term: 6 months Long-term: median 58 months (range, 40–78) UGIS	Short-term recurrence(10% attrition): 24 vs. 9% (sutured vs. mesh) Long-term recurrence (44% attrition): 59 vs. 54% (sutured vs. mesh)	Yes	1b
								2b
Ringley et al. (24)	Prospective case–control	*n* = 44 Size of hiatal defect ≥5 cm BMI significantly higher in the HACD group	HACD	Suture repair with #0 silk sutures, *n* = 22 U-shaped 4 cm × 8 cm mesh anchored with #2–0 silk sutures Nissen fundoplication	12 months UGIS	9 vs. 0% recurrence in favor of HACD 100% (suture repair) vs. 68% (mesh repair) of patients subjected to UGIS Duration of follow up 9.5 months (suture repair) vs. 6.7 months (mesh repair)	Yes	4
Wisbach et al. (25)	Retrospective case series	*n* = 11 Median age 41 years (range 26–60) Hiatal defect >5 cm Recurrent, *n* = 7	HADM	Suture repair with interrupted #0 polyethylene Y-shape mesh sutured with #2–0 polyethylene sutures and tacks Additionally square piece of mesh sutured onto the Y-shaped piece Nissen fundoplication	Median 1 year (range 8–19 months) UGIS	Follow up, *n* = 8 One recurrence	None	4
Jacobs et al. (26)	Retrospective case series	*n* = 127	SIS	Suture repair with interrupted #0 non-absorbable sutures Tension-free repair mesh repair, anchored with interrupted #2–0 non-absorbable sutures Nissen fundoplication, *n* = 102 Toupet fundoplication, *n* = 19 No fundoplication, *n* = 6	Median 3.2 years UGIS and/or UGIE	Three recurrences (65% attrition)	NR	4
Lee et al. (27)	Retrospective case series	*n* = 17 Mean age 65 ± 12 years Mean BMI 31 ± 4 kg/m^2^ Large hiatal hernias (4–7 cm) Revisional repairs, *n* = 4	HACD	Suture repair with interrupted #0 polyethylene sutures U-shaped 4 cm × 7 cm mesh anchored with staples and #0 polyethylene sutures Nissen fundoplication Collis gastroplasty, *n* = 1 Wedge fundectomy, *n* = 3	Mean 14.4 ± 4.4 months (range 5–22) UGIS	Two recurrences	Yes	4
St Peter et al. (28)	Retrospective case–control	*n* = 21 Pediatric patients with hernia recurrence	SIS	Sutured repair with # 2–0 silk sutures and esophagopexy with 4 #3–0 silk sutures, *n* = 13 Pantaloon shaped mesh anchored to the diaphragm and the esophagus with #3–0 silk sutures, *n* = 18 With or without fundoplication	Unclear	Recurrence 4/13 vs. 0/18	NR	4
Fumagalli et al. (29)	Prospective case series	*n* = 6 Median age 65 years Primary or recurrent hernia type II-IV and weak crura	SIS	Suture repair with interrupted #2–0 silk sutures U-shaped mesh anchored with staples Nissen fundoplication	12 months UGIS	Three recurrences		4
Lee et al. (30)	Retrospective case series	*n* = 52 Mean age 56.7 years (range 34–74) Mean size of hernia 7.75 cm (range 5–10)	HACD	Suture repair U-shaped mesh 4 cm × 7cm anchored with 4–6 #2–0 silk sutures Nissen fundoplication	Median 16 months (range 12–24) UGIS	Two recurrences	Yes	4
Varela and Jacks (31)	Retrospective case series	*n* = 5 Mean age 65 ± 7 Years Large type III hernia, mean size 5 cm ± 1	HACD	Suture repair with 5 interrupted non-absorbable sutures Circular 4 cm × 8 cm mesh anchored with four non-absorbable sutures to the crura Nissen fundoplication	NR	No short-term mesh-related complications	NR	4
Diaz and Roth (32)	Retrospective case series	*n* = 46 Mean age 60.3 ± 13.9 Mean BMI 30.3 ± 5.3 Hernia size ≥5 cm on UGIS or UGIE	HACD	Suture repair with interrupted non-absorbable sutures U-shaped 5 cm × 8 cm mesh Tension-free, *n* = 3 Collis gastroplasty, *n* = 2 Nissen fundoplication Selectively gastrostomy	Mean 3.6 months UGIS	Two recurrences (44% attrition) One gastric perforation 30 days post surgery Dysphagia for solids 13%	NR	4
Goers et al. (33)	Retrospective case–control	*n* = 89 Mesh repair: type II-IV hernias with thin crura Suture repair: type III hernias	Biologic NS	Suture repair with pledgeted polyester #0 matress sutures, *n* = 33 Pledgeted polyester #0 matress sutures incorporating the mesh, *n* = 56	NR	Residual resting LESP and mean amplitude higher for mesh repair Similar incidence of dysphagia	NR	4
Alicuben et al. (34)	Retrospective case series	*n* = 82 Median age 63 years Type I hernia, *n* = 35 Type II–IV hernia, *n* = 47 Revisional repair, *n* = 6	HACD	Suture repair with pledgeted #0 polyethylene sutures ± relaxing incision (*n* = 10), ±Collis gastroplasty (*n* = 23) U-shaped mesh anchored with #2–0 silk sutures, tacks or fibrin sealant	5–12 months UGIS or UGIE	Three recurrences (16% attrition)	Yes	4
Molena et al. (35)	Case series	*n* = 18 Mean age 68.2 (range 47–76) Mean BMI 29.2 (range 19–44) Type III, *n* = 7 Type IV, *n* = 11 Revision surgery, *n* = 6	Biologic NS	VATS dissection Suture repair with interrupted non-absorbable sutures *U*-shaped biological mesh anchored with fibrin glue and interrupted sutures Nissen or Toupet and gastropexy Sleeve gastrectomy, *n* = 1 Planned laparotomy, *n* = 2	NR	NR	None	4
Schmidt et al. (36)	Retrospective case–control	*n* = 70 Hernia size 1–5 cm in UGIS or UGIE	HACD	Suture repair with #0 silk sutures, *n* = 32 U-shaped mesh anchored with 4–6 #2–0 silk sutures, *n* = 38	12 months UGIS or UGIE	16 vs. 0% recurrence in favor of HACD 0% dysphagia in the mesh group	NR	4
Sharp et al. (37)	Retrospective case–control	*n* = 52 Pediatric patients with hernia recurrence	SIS or HACD	Suture repair, *n* = 26 Mesh repair, *n* = 25	NA	23.1% (suture) vs. 56% (mesh) of patients presented fever, *p* = 0.02 Mean max temperature 37.8 ± 0.7 (suture) vs. 38.6 ± 0.9 (mesh), *p* = 0.002	None	4
Ward et al. (38)	Prospective case series	*n* = 54 Sliding, *n* = 14 Paraesophageal, *n* = 40 Recurrent, *n* = 3	HACD	Suture repair with #0 polyethylene sutures U-shaped 4 cm × 7 cm mesh anchored with 8–10 #2–0 polyethylene sutures	Min. 6 months UGIS	7.4% recurrence 13% attrition	Yes	4
Watson et al. (39)	Double blind RCT	*n* = 126 Herniation of ≥50% of the stomach	SIS	Suture repair, *n* = 43 Ti-mesh, *n* = 42 SIS, *n* = 41 Granderath buttress technique 2–3 cm × 4–5 cm mesh posterior repair anchored with sutures or tacks	6 months UGIE ± UGIS 12-month symptom outcome	Similar dysphagia rates 7.9% (suture) vs. 5.9% (SIS) vs. 0% (Ti-mesh) recurrence (non-significant)	No	2b

*UGIS, barium contrast upper gastrointestinal series; UGIE, upper gastrointestinal endoscopy; LoE, level of evidence; RCT, randomized controlled trial; SIS, small intestine submucosa, HACD, human acellular cadaveric dermis; LESP, lower esophageal sphincter pressure; BMI, body mass index; VATS, video-assisted thoracoscopic surgery*.

*^a^Based on the Oxford Centre for Evidence-based Medicine – Levels of Evidence (March 2009)*.

## Results

### Repair of Traumatic Diaphragmatic Defects

Two case reports and one case series reported on the use of biologic meshes in traumatic diaphragmatic rupture. Four chronic traumatic defects and two acute ruptures were repaired laparoscopically, or with a laparotomy or a combined (thoracotomy and laparotomy) approach using human acellular cadaveric dermis (HACD) or porcine small intestine submucosa (SIS). Two of the repairs were performed in contaminated surgical fields, one due to inflammation of the herniated gallbladder and one due to pleural empyema. No septic complications requiring prolonged hospital stay or reintervention were reported. Chest X-ray in five of these cases did not reveal recurrence within a 6- to 24-month follow-up period.

### Hiatal Hernia Repair with Mesh Augmentation

A plethora studies reporting use of biologic mesh augmentation of the esophageal hiatus have been published since 2003. Most of these are retrospective industry-sponsored cohort studies. Both HACD and SIS meshes have been used, most commonly in a U-shape or a pantaloon fashion, placed in a retroesophageal position with the limbs of the mesh encircling the esophagus. The graft is anchored to the diaphragm and, in some cases, to the esophagus with non-absorbable sutures, tacks, or fibrin sealant, most commonly following suture repair of the crura or in a tension-free bridging fashion. A Collis gastroplasty has also been reported as a lengthening procedure in cases of a short esophagus (27, 32). Although no adverse effects associated with allografts or xenografts have been reported, in a chart review of 51 pediatric patients, Sharp and colleagues found that fever occurred more frequently after mesh repair and this group of subjects presented with a higher mean temperature during their hospital stay (37).

The best available evidence is provided by two well-designed RCTs (21–23, 39). In an industry-sponsored trial, Oelschlager and colleagues assigned 108 patients with paraesophageal hernia to receive either U-shaped SIS or suture repair. The authors found a significant reduction in the incidence of hernia recurrence (24 vs. 9%) at 6 months (21); however, long-term follow-up data (median 58 months, range 40–78) demonstrated no such benefit (22). Although this outcome may be biased by significant attrition (exceeding 20%), the reported recurrence rate for the mesh group remains unacceptably high.

In a recent double blind RCT that was sponsored by a national authority, suture mesh repair was compared with SIS or collagen-coated titanium mesh augmentation of the hiatus(39); similar recurrence rates at 6 months (7.9 vs. 5.9%, respectively) were found in the suture and biologic mesh repair groups, whereas no recurrence occurred in the synthetic mesh group. This finding, however, should be cautiously interpreted in the presence of wide confidence interval (95% confidence interval, 0.24–9.78). Long-term follow-up data of this trial are pending.

Most authors have focused their interest on potential beneficial effects of biologic grafts in paraesophageal hernia. In a cohort study, Schmidt and colleagues compared suture repair and mesh augmentation with HACD in small hernias (1–5 cm as assessed by barium upper gastrointestinal series or esophagogastroscopy) (36). A benefit of mesh repair was demonstrated, as indicated by a reduced recurrence rate (16 vs. 0%) at 1 year and improvement of symptoms of dysphagia.

## Discussion

Limited evidence exists investigating the role of biologic meshes in traumatic diaphragmatic repair. Low quality evidence (Level 4) suggests that this approach is feasible, at least in chronic cases. Biologic meshes have also been used in contaminated surgical fields with favorable results (Level 5). Because of the difficulties randomizing patients in the acute setting and the rarity of this condition, clinicians should be encouraged to publish their experience with biologic meshes in traumatic diaphragmatic rupture.

Level 1b data currently support lower recurrence rates for biologic mesh repair in the setting of paraesophageal hernia in the short term with conflicting evidence, whereas level 2b data support that this outcome benefit is lost in the long term. In a recent systematic review and meta-analysis of randomized and observational studies conducted by our research group, we found a beneficial short-term effect of mesh augmentation of the hiatus using biologic mesh (odds ratio 3.74, 95% confidence interval 0.92–8.98, *p* = 0.003) (40). However, no long-term outcome data were available for meta-analysis. Low quality data (level 4) suggest that patients with hiatal hernia measuring between 1 and 5 cm may benefit from biologic mesh augmentation. Nevertheless, cost-benefit assessment is lacking and the available evidence favoring biologic over synthetic meshes is insufficient.

The impact of type of biologic graft on hernia recurrence remains to be investigated. Further experimental and clinical research is required to assess new biologic implants in hiatal hernia repair. Although current data have shown SIS implants to be associated with high recurrence rates, other biologic materials have not been adequately investigated. Considering the rarity of cases with traumatic diaphragmatic defects, the effectiveness of biologic implants in such situations may be extrapolated from evidence derived from hiatal hernia repair. Future RCTs are required to investigate the role of biologic meshes in both paraesophageal and small hiatal hernias and evaluate their comparative efficacy to synthetic meshes.

## Author Contributions

Conception and design: SA, FK. Acquisition and interpretation of data: SA, FG, RP. Drafting the work or revision for important intellectual content: SA, FG, RP, FK. Final approval: SA, FG, RP, FK. Agreement to be accountable for all aspects of the work in ensuring that questions related to the accuracy or integrity of any part of the work are appropriately investigated and resolved: SA, FG, RP, FK.

## Conflict of Interest Statement

The authors declare that the research was conducted in the absence of any commercial or financial relationships that could be construed as a potential conflict of interest.

## Appendix

### BioMesh Study Group

Ferdinand Köckerling (Chairman), Stavros A. Antoniou, René Fortelny, Frank A. Granderath, Markus Heiss, Franz Mayer, Marc Miserez, Agneta Montgomery, Salvador Morales-Conde, Filip Muysoms, Alexander Petter-Puchner, Rudolph Pointner, Neil Smart, MarciejSmietanski, Bernd Stechemesser undertaken by the BioMesh Study Group.

### AIM

The BioMesh Study Group has set itself the task of identifying how best to use biological meshes for the various indications. The first step toward achieving that goal is to compile systematic reviews of the different indications on the basis of the existing literature. The available literature sources will be evaluated in accordance with the Oxford Centre for Evidence-based Medicine-Levels of Evidence (March 2009). Next, based on the review findings corresponding Statements and Recommendations are to be formulated in a Consensus Conference for the use of biological meshes for the different indications. The findings of the Consensus Conference are then to be summarized for a joint publication. This present publication is part of the project undertaken by the BioMesh Study Group.

## References

[1] Morgan BS Watcyn-Jones T Garner JP. Traumatic diaphragmatic injury. J R Army Med Corps (2010) 156:139–44. 10.1136/jramc-156-03-02 20919612

[2] Disler DG Deluca SA. Traumatic rupture of the diaphragm and herniation of the liver. Am Fam Physician (1992) 46:453–6. 1636561

[3] Barbiera F Nicastro N Finazzo M Lo Casto A Runza G Bartolotta TV The role of MRI in traumatic rupture of the diaphragm. Our experience in three cases and review of the literature. Radiol Med (2003) 105:188–94. 12835642

[4] Zarzavadjian Le Bian A Costi R Smadja C. Delayed right-sided diaphragmatic rupture and laparoscopic repair with mesh fixation. Ann Thorac Cardiovasc Surg (2014) 20 Suppl:550–3. 10.5761/atcs.cr.12.02065 23411838

[5] Matz A Landau O Alis M Charuzi I Kyzer S. The role of laparoscopy in the diagnosis and treatment of missed diaphragmatic rupture. Surg Endosc (2000) 14:537–9. 10.1007/s004640000362 10890960

[6] Desir A Ghaye B. CT of blunt diaphragmatic rupture. Radiographics (2012) 32:477–98. 10.1148/rg.322115082 22411944

[7] Sliker CW. Imaging of diaphragm injuries. Radiol Clin North Am (2006) 44:199–211. 10.1016/j.rcl.2005.10.003 16500203

[8] Haciibrahimoglu G Solak O Olcmen A Bedirhan MA Solmazer N Gurses A. Management of traumatic diaphragmatic rupture. Surg Today (2004) 34:111–4. 10.1007/s00595-003-2662-8 14745609

[9] Furnée E Hazebroek E. Mesh in laparoscopic large hiatal hernia repair: a systematic review of the literature. Surg Endosc (2013) 27:3998–4008. 10.1007/s00464-013-3036-y 23793804

[10] Antoniou SA Antoniou GA Koch OO Pointner R Granderath FA. Lower recurrence rates after mesh-reinforced versus simple hiatal hernia repair: a meta-analysis of randomized trials. Surg Laparosc Endosc Percutan Tech (2012) 22:498–502. 10.1097/SLE.0b013e3182747ac2 23238375

[11] Stadlhuber RJ Sherif AE Mittal SK Fitzgibbons RJ Jr Michael Brunt L Hunter JG Mesh complications after prosthetic reinforcement of hiatal closure: a 28-case series. Surg Endosc (2009) 23:1219–26. 10.1007/s00464-008-0205-5 19067074

[12] Parker M Bowers SP Bray JM Harris AS Belli EV Pfluke JM Hiatal mesh is associated with major resection at revisional operation. Surg Endosc (2010) 24:3095–101. 10.1007/s00464-010-1095-x 20464417

[13] Antoniou SA Koch OO Antoniou GA Pointner R Granderath FA. Mesh-reinforced hiatal hernia repair: a review on the effect on postoperative dysphagia and recurrence. Langenbecks Arch Surg (2012) 397:19–27. 10.1007/s00423-011-0829-0 21792699

[14] Antoniou SA Pointner R Granderath FA. Hiatal hernia repair with the use of biologic meshes: a literature review. Surg Laparosc Endosc Percutan Tech (2011) 21:1–9. 10.1097/SLE.0b013e31820ad56c 21304379

[15] Teicher EJ Madbak FG Dangleben DA Pasquale MD Human acellular dermal matrix as a prosthesis for repair of a traumatic diaphragm rupture. Am Surg (2010) 76:231–2. 20336913

[16] Pulido J Reitz S Gozdanovic S Price P. Laparoscopic repair of chronic traumatic diaphragmatic hernia using biologic mesh with cholecystectomy for intrathoracic gallbladder. Int J Surg Case Rep (2012) 3:349–53. 10.4293/108680811X13176785204472 22643514 PMC3340968

[17] Al-Nouri O Hartman B Freedman R Thomas C Esposito T. Diaphragmatic rupture: is management with biological mesh feasible? JSLS (2011) 15:546–9. 10.1016/j.ijscr.2012.04.011 22584115 PMC3376699

[18] Oelschlager BK Barreca M Chang L Pellegrini CA. The use of small intestine submucosa in the repair of paraesophageal hernias: initial observations of a new technique. Am J Surg (2003) 186:4–8. 10.1016/S0002-9610(03)00114-4 12842738

[19] Strange PS. Small intestinal submucosa for laparoscopic repair of large paraesophageal hiatal hernias: a preliminary report. Surg Technol Int (2003) 11:141–3. 12931295

[20] Johnson JM Carmody BJ Jamal MK DeMaria EJ. Onlay hiatal reinforcement utilizing human acellular dermal matrix: three case series. Surg Innov (2005) 12:239–41. 10.1177/155335060501200309 16224645

[21] Oelschlager BK Pellegrini CA Hunter J Soper N Brunt M Sheppard B Biologic prosthesis reduces recurrence after laparoscopic paraesophageal hernia repair: a multicenter, prospective, randomized trial. Ann Surg (2006) 244:481–90. 10.1097/01.sla.0000237759.42831.03 16998356 PMC1856552

[22] Oelschlager BK Pellegrini CA Hunter JG Brunt ML Soper NJ Sheppard BC Biologic prosthesis to prevent recurrence after laparoscopic paraesophageal hernia repair: long-term follow-up from a multicenter, prospective, randomized trial. J Am Coll Surg (2011) 213:461–8. 10.1016/j.jamcollsurg.2011.05.017 21715189

[23] Oelschlager BK Petersen RP Brunt LM Soper NJ Sheppard BC Mitsumori L Laparoscopic paraesophageal hernia repair: defining long-term clinical and anatomic outcomes. J Gastrointest Surg (2012) 16:453–9. 10.1007/s11605-011-1743-z 22215243

[24] Ringley CD Bochkarev V Ahmed SI Vitamvas ML Oleynikov D. Laparoscopic hiatal hernia repair with human acellular dermal matrix patch: our initial experience. Am J Surg (2006) 192:767–72. 10.1016/j.amjsurg.2006.08.042 17161091

[25] Wisbach G Peterson T Thoman D. Early results of the use of acellular dermal allograft in type III paraesophageal hernia repair. JSLS (2006) 10:184–7. 16882417 PMC3016123

[26] Jacobs M Gomez E Plasencia G Lopez-Penalver C Lujan H Velarde D Use of surgisis mesh in laparoscopic repair of hiatal hernias. Surg Laparosc Endosc Percutan Tech (2007) 17:365–8. 10.1097/SLE.0b013e318123fc49 18049393

[27] Lee E Frisella MM Matthews BD Brunt LM. Evaluation of acellular human dermis reinforcement of the crural closure in patients with difficult hiatal hernias. Surg Endosc (2007) 21:641–5. 10.1007/s00464-006-9117-4 17287920

[28] St Peter SD Ostlie DJ Holcomb GW III. The use of biosynthetic mesh to enhance hiatal repair at the time of redo Nissen fundoplication. J Pediatr Surg (2007) 42:1298–301. 10.1016/j.jpedsurg.2007.03.040 17618902

[29] Fumagalli U Bona S Caputo M Elmore U Battafarano F Pestalozza A Are surgisisbiomeshes effective in reducing recurrences after laparoscopic repair of large hiatal hernias? Surg Laparosc Endosc Percutan Tech (2008) 18:433–6. 10.1097/SLE.0b013e3181802ca7 18936659

[30] Lee YK James E Bochkarev V Vitamvas M Oleynikov D. Long-term outcome of cruroplasty reinforcement with human acellular dermal matrix in large paraesophageal hiatal hernia. J Gastrointest Surg (2008) 12:811–5. 10.1007/s11605-007-0463-x 18181005

[31] Varela JE Jacks SP. Laparoscopic circular biomeshhiatoplasty during paraesophageal hernia repair. Surg Innov (2009) 16:124–8. 10.1177/1553350609336420 19443865

[32] Diaz DF Roth JS. Laparoscopic paraesophageal hernia repair with acellulardermal matrix cruroplasty. JSLS (2011) 15:355–60. 10.4293/108680811X13125733356594 21985724 PMC3183546

[33] Goers TA Cassera MA Dunst CM Swanström LL. Paraesophageal hernia repair with biomesh does not increase postoperative dysphagia. J Gastrointest Surg (2011) 15:1743–9. 10.1007/s11605-011-1596-5 21773871

[34] Alicuben ET Worrell SG DeMeester SR. Impact of crural relaxing incisions, collis gastroplasty, and non-cross-linked human dermal mesh crural reinforcement on early hiatal hernia recurrence rates. J Am Coll Surg (2014) 219:988–92. 10.1016/j.jamcollsurg.2014.07.937 25256373

[35] Molena D Mungo B Stem M Lidor AO. Novel combined VATS/laparoscopic approach for giant and complicated paraesophageal hernia repair: description of technique and early results. Surg Endosc (2015) 29:185–91. 10.1007/s00464-014-3662-z 24969852

[36] Schmidt E Shaligram A Reynoso JF Kothari V Oleynikov D. Hiatal hernia repair with biologic mesh reinforcement reduces recurrence rate in small hiatal hernias. Dis Esophagus (2014) 27:13–7. 10.1111/dote.12042 23441634

[37] Sharp NE Alemayehu H Desai A Holcomb GW III St Peter SD. Fever after redo Nissen fundoplication with hiatal hernia repair. J Surg Res (2014) 190:594–7. 10.1016/j.jss.2014.05.021 24948540

[38] Ward KC Costello KP Baalman S Pierce RA Deeken CR Frisella MM Effect of acellular human dermis buttress on laparoscopic hiatal hernia repair. Surg Endosc (2015) 29:2291–7. 10.1007/s00464-014-3946-3 25318373

[39] Watson DI Thompson SK Devitt PG Smith L Woods SD Aly A Laparoscopic repair of very large hiatus hernia with sutures versus absorbable mesh versus nonabsorbable mesh: a randomized controlled trial. Ann Surg (2015) 261:282–9. 10.1097/SLA.0000000000000842 25119120

[40] Antoniou SA Müller-Stich BP Antoniou GA Köhler G Luketina RR Koch OO Laparoscopic augmentation of the diaphragmatic hiatus with biologic mesh versus suture repair: a systematic review and meta-analysis. Langenbecks Arch Surg (2015) 400:577–83. 10.1007/s00423-015-1312-0 26049745

